# Correspondence between functional scores from deep mutational scans and predicted effects on protein stability

**DOI:** 10.1002/pro.4688

**Published:** 2023-07-01

**Authors:** Lukas Gerasimavicius, Benjamin J. Livesey, Joseph A. Marsh

**Affiliations:** ^1^ MRC Human Genetics Unit, Institute of Genetics & Cancer University of Edinburgh Edinburgh UK

**Keywords:** deep mutational scanning, multiplexed assay of variant effects, mutations, protein stability, variant effect predictors

## Abstract

Many methodologically diverse computational methods have been applied to the growing challenge of predicting and interpreting the effects of protein variants. As many pathogenic mutations have a perturbing effect on protein stability or intermolecular interactions, one highly interpretable approach is to use protein structural information to model the physical impacts of variants and predict their likely effects on protein stability and interactions. Previous efforts have assessed the accuracy of stability predictors in reproducing thermodynamically accurate values and evaluated their ability to distinguish between known pathogenic and benign mutations. Here, we take an alternate approach, and explore how well stability predictor scores correlate with functional impacts derived from deep mutational scanning (DMS) experiments. In this work, we compare the predictions of 9 protein stability‐based tools against mutant protein fitness values from 49 independent DMS datasets, covering 170,940 unique single amino acid variants. We find that FoldX and Rosetta show the strongest correlations with DMS‐based functional scores, similar to their previous top performance in distinguishing between pathogenic and benign variants. For both methods, performance is considerably improved when considering intermolecular interactions from protein complex structures, when available. Furthermore, using these two predictors, we derive a “Foldetta” consensus score, which improves upon the performance of both, and manages to match dedicated variant effect predictors in reflecting variant functional impacts. Finally, we also highlight that predicted stability effects show consistently higher correlations with certain DMS experimental phenotypes, particularly those based upon protein abundance, and, in certain cases, can significantly outcompete sequence‐based variant effect prediction methodologies for predicting functional scores from DMS experiments.

## 1 INTRODUCTION

Recent decades have seen massive breakthroughs in optimizing genomic sequencing for large‐scale operations, revealing the high prevalence of genetic variation in human populations (Dunham et al., 2012; Karczewski et al., 2020). Many genetic variants are from missense mutations, which cause a change in the identity of single amino acid residues at the protein level (Landrum et al., 2014). However, the precise phenotypic consequences of most variants remain uncertain as mutants are seldom functionally characterized in clinical settings, while alternative approaches to conclusively classify variants, such as genetic testing and pedigree studies, require many cases (Iversen Jr et al., 2011).

Multiplex assays of variant effects (MAVEs) have emerged as methodologies with the potential to measure the effects of large numbers of genetic variants in parallel within a single experiment (Fowler & Fields, 2014; Starita et al., 2017). MAVEs produce interpretable variant‐function maps by associating each variant to a quantitative assay measurement for a select phenotype. MAVEs that involve protein‐based assays of amino acid variants are often referred to as deep mutational scanning (DMS) experiments. DMS has been widely adopted to explore effects of amino acid variation using a variety of different experimental phenotypes, such as protein abundance, activity or general cellular fitness (Fowler & Fields, 2014). However, while the number of proteins that have been characterized through DMS grows constantly (Esposito et al., 2019; Kuang et al., 2021), and use of these methodologies and coordination between groups is increasing through the Atlas of Variant Effects Alliance (AVE Alliance Founding Members, 2021), as of now, DMS is not up to the challenge of evaluating all possible substitutions in the entire proteome, both due to costs and the inherent limitations of assaying specific phenotypes.

As an alternative or complement to experimental approaches for characterizing variants, considerable efforts have been put into developing generalizable computational models for predicting the effects of protein variants. A large number of variant effect predictors (VEPs) exist that leverage different properties, including evolutionary sequence conservation, phylogenetic relationships and physicochemical properties, to evaluate the likelihood of a variant being damaging (Livesey & Marsh, 2022a). However, the scores output by these VEPs seldom provide an interpretable context to the underlying disease mechanism. An alternative approach is presented by structure‐based protein stability predictors, which can evaluate the change in Gibbs free energy of folding (ΔΔG) or intermolecular interaction upon mutation (Iqbal et al., 2021). Stability predictors are frequently used in the fields of protein engineering and even clinical genetics, despite not being trained for disease identification, because they can distinguish between stabilizing and destabilizing energetic effects and provide clues as to possible pathogenic mechanisms (Gerasimavicius et al., 2020).

The methodological approaches to predicting stability impacts of mutations are diverse. FoldX (Delgado et al., 2019) and Rosetta (Alford et al., 2017) use empirical physics‐based potentials with additional statistical terms based on observations from bimolecular structures. ENCoM is a unique method that takes into account how mutations can impact protein dynamics and stability through normal mode analysis (Frappier et al., 2015). A combination of evolutionary and structural information has been employed in the untrained DDGun3D predictor (Montanucci et al., 2022). Some recent predictors, like mCSM, have been derived through machine learning using various features (Pires et al., 2014). Given such heterogeneity in approaches, numerous studies have been carried out to benchmark the performance of these predictors in reproducing realistic ΔΔG values that agree with experimental thermostability data (Caldararu et al., 2021; Iqbal et al., 2021; Khan & Vihinen, 2010; Lonquety et al., 2007; Marabotti et al., 2021; Pancotti et al., 2022; Potapov et al., 2009). Furthermore, there have been attempts to explore and address biases, such as data circularity, overprediction of destabilizing variants and lack of prediction symmetry (König et al., 2016; Montanucci et al., 2022; Montanucci, Savojardo, et al., 2019; Pucci et al., 2018; Usmanova et al., 2018). However, as stability predictors are now routinely used for protein engineering and disease identification purposes (Blanco et al., 2018; Fu et al., 2022; Heyn et al., 2019; Holt et al., 2019; Othman et al., 2020; Williamson et al., 2020; Yu et al., 2022; Zhao et al., 2021), it is crucial to know how well ΔΔG serves as a proxy score for pathogenicity, and thus how prevalent are destabilizing loss‐of‐function mechanisms in the pool of all possible mutations (Birolo et al., 2021; Gerasimavicius et al., 2022; Sanavia et al., 2020). We have previously assessed the performance of ΔΔG values from stability predictors in distinguishing between pathogenic and putatively benign missense variants in a classification task (Gerasimavicius et al., 2020). Phenotypic assays now offer further opportunity to more quantitatively interrogate the extent to which predicted ΔΔG agrees with assayed fitness or activity of protein variants.

DMS datasets currently provide the most extensive experimentally derived representation of the functional variant effect landscape, and they have been very successfully utilized in recent VEP benchmarking studies (Livesey & Marsh, 2020; Livesey & Marsh, 2022b). It was further shown that DMS assay values themselves can, in some cases, be better at distinguishing pathogenic from benign variants than current computational approaches. However, a number of caveats should be understood when using DMS scores to evaluate how damaging effects are represented through predicted changes in stability. Realistically, we can expect that assays that evaluate phenotypes such as protein abundance or complex formation, should show the best agreement with stability predictions, as they are well suited to detect destabilizing loss‐of‐function molecular mechanisms. Other types of assays, such as general competitive growth experiments, are potentially sensitive to non‐destabilizing but damaging mutations, such as those associated with gain‐of‐function or dominant‐negative effects. We have previously shown that damaging mutations, which manifest through such non‐loss‐of‐function mechanisms, tend to be mild at a protein structural level, and not well identified through prediction of stability effects (Gerasimavicius et al., 2022). Thus, some specific types of assays, which do not measure stability directly, may show very heterogenous agreement with stability predictors. However, we believe DMS values provide an unbiased, independent way of comparing predictors, and at the same time allow us to explore how well destabilizing loss‐of‐function mechanisms can be identified through specific experimental phenotypes.

In this study, using a large number of DMS datasets as a benchmark, we quantified the capability of structure‐based protein stability predictors to accurately rank the functional impacts of variants. We demonstrate that FoldX and Rosetta predictions derived on protein complex structures significantly outperform other tools in assessing the functional impact of mutations. We also show how evaluating full biological assemblies improves our ability to relate predictions to functional phenotypes involving protein or DNA binding. Interestingly, we find that combining the predictions from FoldX and Rosetta into a consensus “Foldetta” score considerably improves the correlation with DMS data, especially when using full complex structures, leading to a performance that matches dedicated variant effect predictors. Finally, we explore how certain types of DMS phenotypes, specifically ones related to protein abundance, correlate the best with variant stability predictions due to their closer association with destabilizing loss‐of‐function mechanisms.

## 2 RESULTS

### 2.1 Considerations of stability predictor benchmarking with DMS datasets

For this study, we gathered 49 different DMS datasets for 39 unique protein targets. A majority of the datasets we used were collected and outlined previously in the comprehensive VEP benchmarks from Livesey and Marsh (2020); Livesey and Marsh (2022b). This mostly included assays of human proteins, but also experiments on yeast, bacterial, and viral proteins. In addition, new datasets have been subsequently released and published in the MaveDB (Esposito et al., 2019), which were combined with previous data to form our benchmarking dataset. The full list of genes and DMS datasets used in this study is available in Table S1. For the additional DMS experiments that contained fitness values from assays under multiple conditions, we selected the option most representative of native‐like conditions, for instance grown under no additional treatments than were required by the phenotypic assay or experimental setup.

The DMS datasets used are heterogenous in terms of the phenotypes that were assayed for. The most frequent category involves human gene complementation of native genes in yeast, with the fitness scores being derived from competitive growth of variants in deep mutational scanning experiments. VAMP‐seq is another methodology, which interrogates the abundance of GFP‐fused variant proteins and thus also their stability (Matreyek et al., 2018). Other methods include toxicity assays, assessing changes to binding strength through two‐hybrid or phage display assays, or activity assays tailored to specific targets, for instance patch‐clamp assessment of cell currents for KCNQ4 potassium channel variants (Zheng et al., 2022). In this work, for cases where a single gene has multiple associated DMS datasets, we identify the datasets alphabetically, surrounded by parentheses, for example, BRCA1(a) and BRCA1(b) represent two independent DMS experiments performed by different groups using different functional assays.

Different DMS datasets for the same genes can show variability in variant impact values due to different experimental conditions or even the phenotypes being assayed, however, they generally show moderate to high Spearman's correlations, averaging at around 0.66, suggesting that they represent a sufficiently robust approach to benchmark variant effect prediction performance (Livesey & Marsh, 2020). Mutations that excessively perturb the stability of a protein and lead to its degradation ought to be reflected by most assay types. However, we can also imagine that many DMS assays would be sensitive to mutations that affect the protein in some way other than loss of function due to intra‐ or inter‐molecular destabilization. Thus, the degree of agreement between ΔΔG values and DMS scores should also tell us something of the pervasiveness of destabilizing loss‐of‐function mechanisms for a given gene or tested phenotype.

Most of the stability predictors we use here depend on structural inputs. We therefore derived a structural variant map, linking each variant in every DMS dataset to a residue within a protein structure from the Protein Data Bank (Berman et al., 2000), using the same strategy as described recently (Gerasimavicius et al., 2022). AlphaFold2 models were used for proteins or select residues in infrequent cases where they were not covered by experimental structures. It has been recently demonstrated that accurate stability predictions can also be delivered using modeled protein structures, with AlphaFold2 providing suitable inputs for FoldX even for proteins without homologs in the training set (Akdel et al., 2021; Blaabjerg et al., 2023; Pak & Ivankov, 2022).

We tested nine stability predictors, seven of which we have previously also explored for their ability to distinguish between pathogenic and putatively benign human variants (Gerasimavicius et al., 2020). On top of FoldX, Rosetta, INPS3D, PoPMuSiC, mCSM, ENCoM, and DynaMut2, we have included DDGun3D, an “untrained” stability prediction method, as well as the recently released RaSP which offers rapid evaluation of variants based upon sequence alone through a neural network model (Alford et al., 2017; Blaabjerg et al., 2023; Dehouck et al., 2011; Delgado et al., 2019; Frappier et al., 2015; Montanucci et al., 2022; Pires et al., 2014; Rodrigues et al., 2021; Savojardo et al., 2016). While most methods only offer functionality of evaluating stability perturbing effects of mutations on monomeric structures, FoldX, ENCoM and Rosetta were also evaluated in terms of full protein complex structures, if they were available, as this functionality is easily accessible in these predictors. Finally, we not only explored the agreement of ΔΔG values, which range from stabilizing to destabilizing, but also absolute change in stability, |ΔΔG|, as a metric of general energetic perturbation of the structure. We have previously shown that considering strongly stabilizing variants as deleterious through use of |ΔΔG| improves the disease identification performance of most stability predictors, likely due to stability predictors sometimes mispredicting the sign of the ΔΔG, and stability‐increasing mutations occasionally being pathogenic (Gerasimavicius et al., 2020).

**FIGURE 1:**
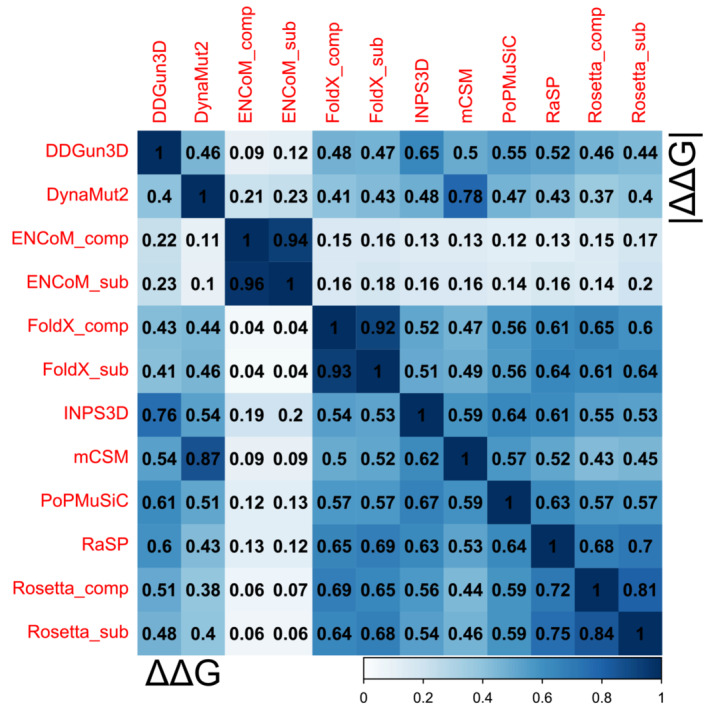
Most protein stability prediction methods display moderate agreement with each other. The colored scale bar represents the absolute magnitude of Spearman's rho values from pairwise comparisons calculated for complete observations, calculated across variants pooled from all the genes. The lower matrix triangle shows Spearman's correlation calculated using the raw Gibbs free energy values, taking into account both destabilizing and stabilizing effects, while the upper triangle contains rho values derived using only the absolute magnitudes. All predictor ΔΔG values were adjusted to match for stabilizing and destabilizing effect directions.

Before benchmarking the predictors on DMS measurements, we first compared the agreement between all predictors for the full dataset of explored variants to get a sense of the heterogeneity between different methodological approaches. We calculated pairwise Spearman's rho values across all evaluated variants between each predictor pair, using both ΔΔG and |ΔΔG| metrics (Figure 1). Without taking the high correlation between monomeric vs complex predictions for the same predictor into account, or DynaMut2 and its methodological component mCSM, we see that, overall, most of the predictors also show fairly good agreement with each other, with the average absolute Spearman's rho value for ΔΔG at 0.49, rising to 0.60 if we exclude ENCoM.

ENCoM predictions stand out as the most unrelated to any other predictor, with the highest correlation for ΔΔG being with DDGun3D at 0.23. Rosetta predictions appear to be the most divergent in terms of comparing monomeric vs complex values for the same mutations, likely as a result of the energetic minimization procedure of structures, treating complexes as a single chain. As in our previous work, using absolute stability values did not prove beneficial to increase the agreement between most predictors, with the exception for ENCoM correlations with FoldX and Rosetta. We have also previously demonstrated that ENCoM benefits the most from use of absolute values for classification tasks, as it tends to overpredict stabilizing effects. The highest correlations of 0.76 were observed between DDGun3D and INPS3D, as well as 0.75 between RaSP and Rosetta. Indeed, one would expect RaSP and Rosetta to show a high degree of correlation, as RaSP has been trained not on experimental ΔΔG values, but on Rosetta predictions (Blaabjerg et al., 2023). The high correlation between DDGun3D and INPS3D is likely due to the fact that both methods are essentially extensions of their underlying sequence and alignment‐based models, and therefore both directly utilize information about evolutionary sequence conservation, in contrast to all other predictors.

### 2.2 Correspondence between stability predictions and DMS values

**FIGURE 2:**
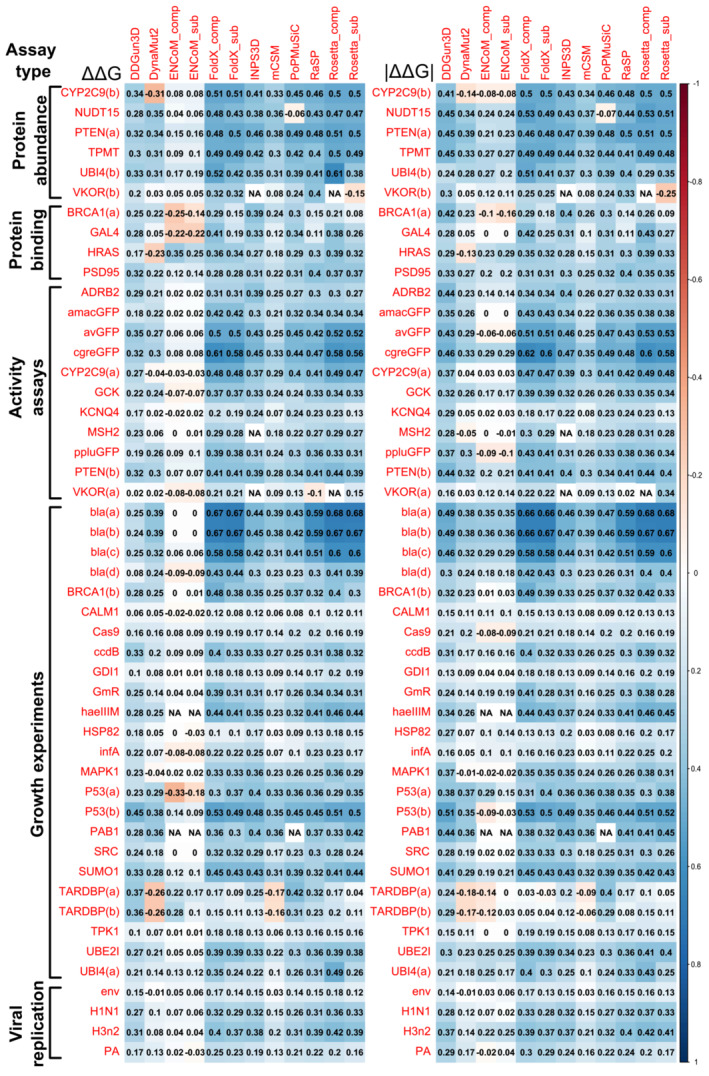
Stability predictor scores can correlate highly with scores from DMS experiments, but performance is highly heterogenous. The colored scale bar represents the absolute magnitude of Spearman's rho values from pairwise comparisons calculated for complete observations. All predictor ΔΔG values were adjusted to match for stabilizing and destabilizing effect directions.

For each stability predictor and DMS dataset pair, we calculated Spearman's correlations using the shared subset of observations (Figure 2). Both the predictor and DMS score directions were adjusted for consistency (i.e., higher ΔΔG values indicate increased destabilization and higher DMS scores indicate more damaging effects). While we observed that most predictors rank DMS dataset variants with moderate consistency between each other, the overall correlations tend to be fairly low, with the average rho values throughout the benchmark at 0.26 and 0.28 for ΔΔG and |ΔΔG|, respectively. This is in‐line with the recent observations from Høie et al. showing an average Spearman's correlation value of 0.25 between Rosetta ΔΔG and a number of DMS datasets (Høie et al., 2022). Overall, even direct experimental thermostability values and computational predictions are often shown to only correlate on the order of ~0.5, with some works showing that the practical upper bound for such comparisons can only reach ~0.8 due to experimental data quality (Montanucci, Martelli, et al., 2019).

However, other works have shown individual correlations between stability predictions and DMS functional scores can reach up to 0.57 for targets like NUDT15 (Cagiada et al., 2021). In our analysis, Rosetta and FoldX stability predictions for the *E. coli* beta lactamase (bla) antibiotic resistance DMS datasets bla(a) and bla(b) produced the highest observed correlations in this benchmark, ranging between 0.66 and 0.68, while the best correlation for bla(d), a different antibiotic resistance experiment, was only 0.44. Rosetta complex ΔΔG values also appear to correlate well (0.61) with dataset UBI4(b), based on a FACS assay that relates well to stability. The next best correlating datasets for other genes had Spearman's rho values of up to 0.53, across both ΔΔG and |ΔΔG|.

We have previously shown that dominant‐negative and gain‐of‐function disease variants tend to be structurally milder and demonstrate weak stability perturbation (Gerasimavicius et al., 2022). As such, they are harder to distinguish from benign variants through stability prediction. This is likely to contribute to the low correlations observed for CALM1, TARDBP and SRC datasets, which are known to also be associated with non‐loss‐of‐function disease mechanisms, such as the dominant‐negative effect in CALM genes (Badone et al., 2018; Boczek et al., 2016; Rocchetti et al., 2017), and gain‐of‐function mutations in TARDBP (Kabashi et al., 2010; Suk & Rousseaux, 2020) and SRC (Abe et al., 2019; Turro et al., 2016).

ENCoM, the method with weakest correlations overall, leverages a unique prediction approach based on normal mode analysis, which is purported to take into account variant effects on protein dynamics. However, it also benefits the most out of all predictors from the use of absolute stability values as a metric, increasing Spearman's rho by up to 0.36, compared to correlations achieved using raw stability values. This approach shifts the perspective toward evaluating mild vs perturbing effects, instead of stabilizing vs destabilizing, suggesting a likely tendency to overpredict variants as stabilizing. This also appears to affect DDGun3D to a lesser extent.

**FIGURE 3:**
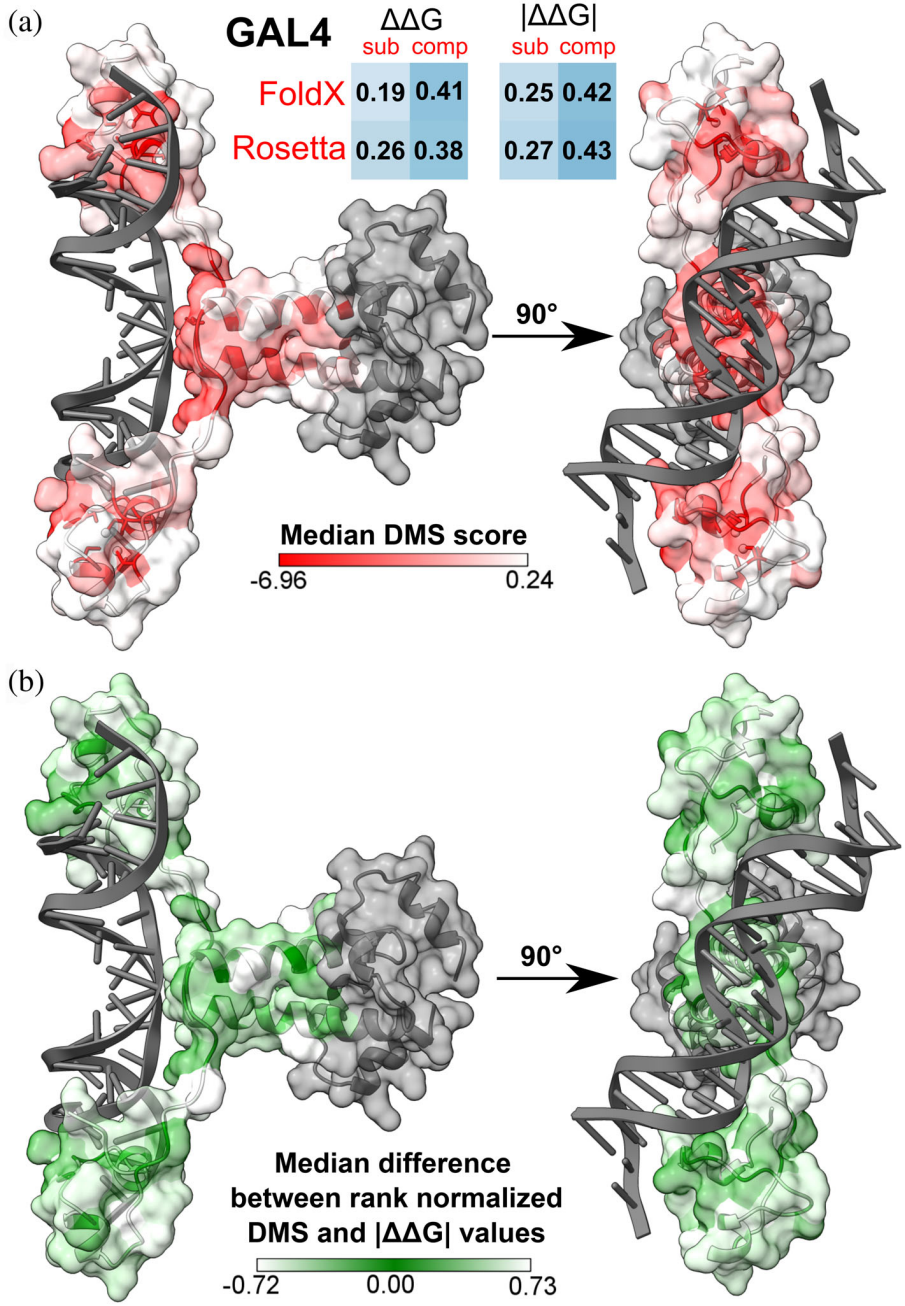
Stability predictors that can account for intermolecular interactions between proteins and other molecules, such as DNA, allow to explain a larger extent of functional variant effects through the scope of stability. (a) Values in squares represent Spearman's correlations for GAL4 variants between predicted Gibbs free energy changes and DMS scores, in the case of using just one subunit structure, or evaluating stability on the entire complex assembly. The DMS dataset for GAL4 contains variant enrichment scores from a yeast two‐hybrid experiment. Protein structures are colored based on the per‐position median variant score, with lower values indicating more functionally sensitive positions, and gray indicating missing values. GAL4 variants used for the analyses were mapped to PDB structure 3COQ, containing a GAL4 dimer and a DNA double‐strand. (b) Protein structures are colored based on the agreement between rank normalized DMS scores and absolute FoldX ΔΔG predictions. Both the DMS and FoldX scores were rank normalized to the range of 0–1, where a positive delta for a variant indicates FoldX is underpredicting its functional effect, in relation to DMS data, while a negative delta means FoldX overestimates the functional impact of a variant. A value close to zero (green) indicates agreement between FoldX and the DMS assay on the relative severity of a variant.

Importantly, we also explored how taking into account intermolecular interactions, by using complex structures, impacts variant stability prediction, and in turn the evaluation of functional effects. We compared results from FoldX, Rosetta and ENCoM, which were the only methods that allowed easy use of biomolecular complex structures in prediction. This led to considerable improvements in the agreement with DMS measurements in certain cases. For example, both FoldX and Rosetta showed marked increases in correlation with GAL4 DMS values when the full DNA‐bound structure was utilized, as opposed to just the monomeric subunit in isolation. This is illustrated in Figure 3a, where the structure of the GAL4 complex is shown colored based on DMS values, showing that hotspots of highest functional variant impact appear not only at the protein dimer interface, but also at interaction sites with DNA. Such mutation effects at interfaces would be missed by stability predictions that do not take intermolecular interactions into consideration. If we repeat the visualization (Figure 3b), this time coloring the residue positions by agreement between the severity of DMS scores and absolute FoldX stability predictions (see Section 4), we can see that many of the accurately evaluated positions in green overlap with the DMS score hotspots in Figure 3a, suggesting that the most damaging variants that disrupt DNA binding may be predicted the best. However, FoldX also appears to under‐ and over‐predict other functional variant outcomes, on a per‐position and per‐mutation basis, either due to DMS scores being affected by other molecular dysfunction mechanisms or the inaccuracy of the FoldX methodology itself.

### 2.3 Relative ranking of stability predictor performance in assessing variant functional impacts

**FIGURE 4:**
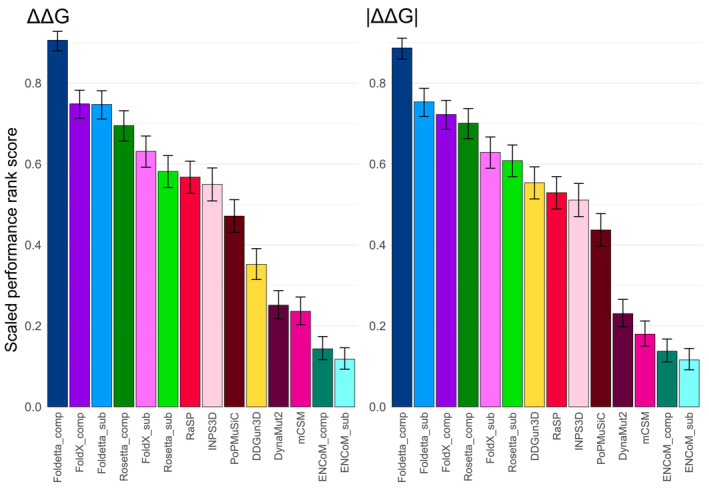
Complex‐based FoldX and Rosetta stability predictions are significantly more accurate at ranking variant effects in relation to DMS scores than other stability evaluation tools. Computational protein stability predictor rankings were derived based on comparisons of pairwise correlations with DMS scores, see “Section 4.” Error bars denote the 95% confidence interval of a binomial test.

To compare the relative performance of our tested predictors, we used a scoring and ranking scheme based on pairwise predictor correlation comparisons on each DMS dataset, as recently introduced (Livesey & Marsh, 2022b). Each predictor would be given a point for demonstrating a higher Spearman's rho value for a DMS set against another prediction tool, or both would get half a point for a tie. The advantage of this ranking strategy is that it accounts for the fact that not all methods successfully made predictions for all mutations in all proteins, as the pairwise comparisons are performed only on the shared variant subset that both predictors were successfully able to evaluate. In the end each, predictor's score was scaled by the total number of successful comparisons it had been involved in, resulting in a relative performance ranking, for both ΔΔG and |ΔΔG| (Figure 4). Given the top performance of FoldX and Rosetta in Figure 2, we also compared a combined approach we termed “Foldetta,” which represents the mean of FoldX and adjusted Rosetta predictions (rescaled from REUs to kcal/mol, see Section 4).

Using either ΔΔG or |ΔΔG| values results in very similar rankings overall. If we consider only the original predictors, in both cases, FoldX and Rosetta values derived on protein complexes were the top performers by a large margin. However, combining complex‐based scores from the two tools produces a superior predictor, with the Foldetta_comp ensemble coming out ahead by ~0.15 points in the ranking. Rankings starting at seventh place change slightly depending on whether we are using ΔΔG or |ΔΔG| values with DDGun3D seeing a large performance increase when using absolute scores. However, in both cases the four methods that show the least correspondence with DMS rankings are DynaMut2, mCSM, ENCoM for complexes and ENCoM for monomers. The surprising performance increase when taking into account the consensus of FoldX and Rosetta predictions contrasts sharply with multi‐feature or consensus methods like DynaMut2, DDGun3D and INPS3D, but demonstrates the utility of even simple untrained ensembling when combining the right inputs.

Although a comprehensive benchmarking of VEPs using DMS datasets has been recently carried out (Livesey & Marsh, 2020), we decided to compare how well predictors, which have been intentionally derived for functional variant effect evaluation, agree with experimental DMS scores on our structural benchmarking dataset. Many of our DMS dataset genes are not of human origin, and many VEPs are not designed to produce predictions for nonhuman proteins. We thus picked a small selection of methodologically diverse methods that had produced a sufficient number of predictions for most DMS datasets. This included two simple substitution matrices as baseline methods for assessing amino acid similarity, the widely used classical method SIFT (Ng & Henikoff, 2003), more modern methods PROVEAN(Choi & Chan, 2015) and SNAP2 (Hecht et al., 2015), as well as state‐of‐the‐art predictors DeepSequence (Riesselman et al., 2018) and EVcouplings (Hopf et al., 2019). Figure S1 shows that, compared to stability predictors, VEP correlations are considerably higher and more consistent across the different gene datasets. They achieve average Spearman's correlations of 0.36, and 0.42 if we exclude the BLOSUM62 and Grantham substitution matrices (Grantham, 1974; Henikoff & Henikoff, 2000)—considerably higher than the average value of ~0.26 observed for stability predictors. If we include VEPs in our ranking scheme, we see that FoldX and Rosetta outperform the substitution matrices and SIFT, but cannot perform as well as modern VEPs for predicting functional scores from DMS experiments (Figure S2), consistent with our previous observation that stability predictors are, overall, less useful than VEPs for the identification of pathogenic missense mutations (Gerasimavicius et al., 2020). Interestingly, the combined Foldetta score shows substantial capacity to accurately reflect the relative functional impacts of variants, comparable to the top tested VEPs—EVcouplings and SNAP2.

### 2.4 Computational stability predictions show better agreement with DMS scores derived through protein abundance‐based assays

We have observed that the highest heterogeneity in correlation values arises not between different predictors, but across DMS datasets. We note that phenotypes that tend to correlate well with stability prediction values come from VAMP‐seq or other fluorescence‐based experiments (e.g., NUDT15, PTEN(a), TPMT, CYPC9(a), UBI4(b)). While growth assay datasets from some specific targets, like bla or P53, also show good agreement, they are uncharacteristic and more likely represent good protein‐specific performance unrelated to the underlying assay type. Abundance‐based approaches could be said to directly relate to the stability of target proteins, thus being better tailored to detecting loss of protein function. VAMP‐seq has been specifically developed for this purpose and shown to correlate well with experimental thermodynamic and predicted stability measures (Matreyek et al., 2018; Suiter et al., 2020). On the other hand, competitive growth assays produce fitness values that relate to numerous underlying effects and various molecular mechanisms and show mixed tendencies for both high and low correlations, depending on the target.

To more quantitatively explore whether any specific assay types stand out in their agreement with stability prediction values, we classified the DMS datasets broadly into five groups, as outlined in Table S3, depending on the phenotype assaying approach. Abundance‐based assays represent experiments that make use of VAMP‐seq and other fluorescent‐tagging approaches that can quantify protein expression and stability in cells, being able to directly assess whether variants lead to a loss‐of‐function through degradation. Growth experiments involve mutant competition or antibiotic survival and are able to characterize functional effects from multiple molecular mechanisms. Binding assays such as phage display or two‐hybrid experiments can represent variant impacts on intermolecular interactions, which ought to relate well to stability predictions for tools that can take into account relevant complex structures. The activity assay category includes target‐specific experimental setups more directly measuring the functional mutant effects, and not just through the proxy of growth or protein stability. We separated out viral protein datasets, based on replication assays, into a separate category due to them being quite dissimilar from all other proteins.

**FIGURE 5:**
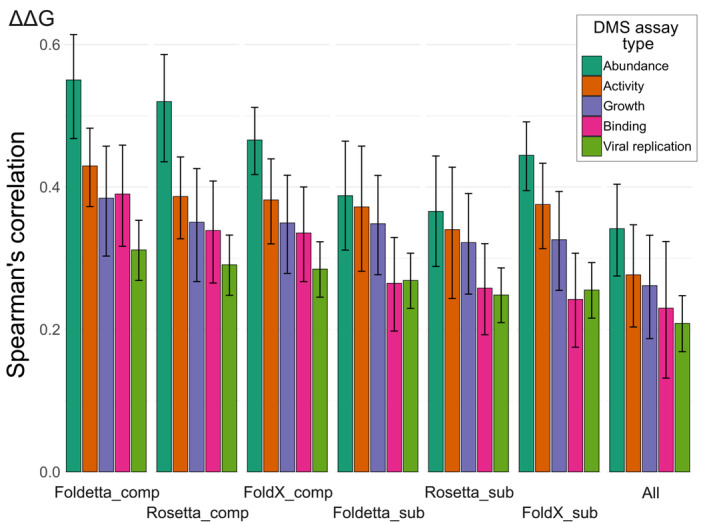
Stability predictions correlate best with results from assays interrogating protein abundance. Correlations were calculated per‐dataset, and the values shown represent assay group means. Error bars denote the 95% confidence interval, found by using the Fisher z transform of the correlation. Only raw ΔΔG values from stability predictors were used in the comparison.

Figure 5 shows the mean per‐dataset Spearman's correlations with the top‐ranking stability predictors FoldX, Rosetta, their combined Foldetta score, as well as the mean across all methods, for each assay phenotype group. The trend clearly demonstrates that stability predictors best reflect functional scores from abundance‐based DMS datasets, both in the case of only the best methods, as well as for all predictors, while viral replication datasets show the worst agreement. Activity‐based assays show good agreement mostly due to high correlations on the GFP datasets, which are bound to be more representative of loss‐of‐function mechanism mutations. Competitive growth assays, which are a popular generalized approach, appear to show mixed agreement on a per‐gene level, but are not well correlated with changes to stability overall, possibly owing to destabilizing loss‐of‐function mechanisms being more prominent only in certain genes. Curiously, half of the binding‐based assays do not show good agreement even with FoldX or Rosetta, which are able to evaluate interactions in complex structures, or show a marked improvement between predictions derived on monomeric structures vs fullest available structure.

We investigated the coverage of mapped and other available structures for the binding DMS proteins (PSD95, HRAS, GAL4, BRCA1), to see what factors might underly the low correlations. We found that structures for HRAS (PDB IDs: 2CE2 and 6P0Z) and PSD95 (PDB ID: 6QJL) did not contain the binding partners relevant for the specific DMS assays, or were monomeric, explaining the inability of stability predictors to reflect intermolecular interactions. More suitable complex structures were not currently available in the PDB. However, both GAL4 and BRCA1(a) DMS datasets show increased correlation with stability values, when using complex structures. The GAL4 structure (PDB ID: 3COQ) contains a transcription factor dimer that is bound to a DNA duplex, allowing us to evaluate variant effects both on dimerization and DNA recognition. The BRCA1(a) dataset was derived from an assay interrogating BRCA1‐BARD1 heterodimer interactions, which are represented in the structural data (PDB IDs: 7JZV and 1JM7).

**FIGURE 6:**
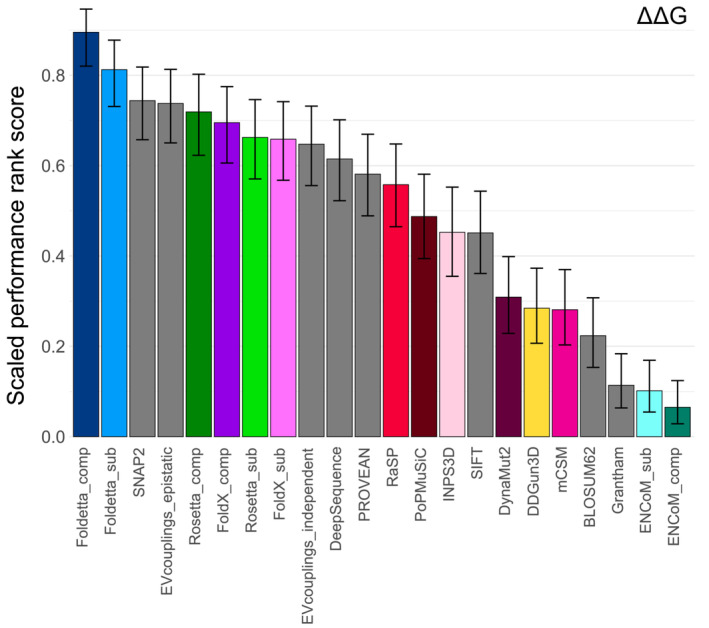
Computational stability predictors can outcompete top VEPs when assessing agreement only against abundance‐based DMS data. Computational protein variant effect predictor rankings were derived based on comparisons of pairwise correlations against DMS scores, see “Section 4.” Only raw ΔΔG values from stability predictors were used in the comparison. Error bars denote the 95% confidence interval of a binomial test.

Considering the strong correlation differences that may arise based on assay type, we decided to investigate how the ranking is affected by only using DMS datasets that have been shown to be well reflected by stability predictions. We explored DMS scores from experiments on protein abundance, which include VAMP‐seq experiments and other fluorescence‐based approaches. In our comparison we also included VEPs to see how stability predictors compare on DMS datasets that are best suited for loss‐of‐function prediction. Figure 6 shows a considerably steeper rank distribution, strongly establishing the combined Foldetta score not only as the approach most reflective of functional assay scores among stability‐based methods, but also as the overall best predictor, significantly outperforming all VEPs in the case when predictions from complex structures are used against abundance‐based DMS scores. Compared between each other, complex‐based FoldX and Rosetta demonstrate similar performance on DMS experiments based on abundance phenotypes, and, judging by the overlapping confidence intervals, show a competitive performance with top VEPs on these datasets. While this current generation of stability predictors are not likely to be particularly useful in the direct identification of pathogenic variants on the proteome scale, compared to state‐of‐the‐art VEPs, they offer a clear advantage over VEPs in terms of interpretability of mechanistic effects, especially in this case where we see that raw stability predictions correlate better than absolute values with functional DMS scores, indicating the distinction between stabilizing and destabilizing variants is important for accuracy. This contrasts with our past results for stability predictor performance in a classification task between pathogenic and benign variants, where |ΔΔG| values, focusing on the overall magnitude of a mutation's impact on stability, showed better predictivity. More importantly, the performance increase we observed when combining the scores from our two best methods by simple averaging hints that there are ample opportunities to further increase the capability of stability predictors when identifying functionally impactful variants and identifying putative disease mutations.

## 3 DISCUSSION

Our study primarily aimed to examine the relationship between stability, structure and function, by interrogating the capacity of different protein stability predictors to score variants in line with experimentally determined functional impacts. We found that FoldX and Rosetta show the strongest correlations with DMS measurements, and also demonstrated the importance of protein complex structures for evaluating the functional impact of variants to the fullest extent. DMS experiments proved to be instrumental for this benchmarking task, as their results are currently the most direct representation of the functional protein variant landscape. Our results support the choice of Rosetta as a predictive tool, which has recently been used in similar studies exploring DMS functional score relationships with stability‐ and conservation‐based metrics (Cagiada et al., 2021; Høie et al., 2022). Unlike many VEPs, which are optimized for human mutations, or influenced by the widely varying sequence coverage across evolutionary space, stability predictors should be well suited to evaluate proteins in an organism‐agnostic manner, as most are grounded in physics or approximate physical terms by proxy. Finally, we must accentuate that stability predictors are not designed for disease variant identification, and their training and design is aimed at reproducing realistic ΔΔG values that reflect experimental thermostability changes, and not at ranking functional impacts. Thus, our predictor ranking does not necessarily imply that FoldX or Rosetta are the most accurate tools for predicting either the ΔΔG magnitude, or the direction of stability perturbing effects. Despite this, stability predictors are still routinely used in clinical research for variant prioritization and mechanistic interpretation (Heyn et al., 2019; Holt et al., 2019; McEntagart et al., 2016; Williamson et al., 2020), mostly because of their high interpretability due to their structure‐based nature. For instance, evaluation of a set of clinically severe Pax6 mutations using FoldX revealed that many are insufficiently destabilizing to disrupt the native DNA interactions, and instead, suggested a gain‐of‐function off‐target mechanism behind the worse‐than‐null phenotypes (Williamson et al., 2020). Similarly, dominant mutations in Itpr1, a homo‐tetrameric ion channel protein, were found to be insufficiently destabilizing to disrupt complex formation, suggesting a dominant‐negative mechanism of action, with mild mutations in a single subunit causing complex poisoning (McEntagart et al., 2016). Exploring the ability of ΔΔG to reflect functional impact could lead to a more effective application of these methodologies for such purposes.

We have previously also demonstrated FoldX was the best out of all tested stability predictors at distinguishing between confirmed human disease variants and putatively benign ones through both ΔΔG and |ΔΔG|, while Rosetta did not rank as high on that particular task (Gerasimavicius et al., 2020). One aspect of FoldX performance that made it useful for disease identification was its tendency to assign excessively large stability perturbation scores to disease variants, due to the clashes they caused within structures, very effectively separating the score densities between putatively benign mutations and truly deleterious ones. However, in this current work we demonstrate that FoldX and Rosetta also have the capacity to maintain an accurate relative ranking of functional variant effects, and not just produce outlier scores for disease mutations. This is potentially of great benefit, as it shows such predictors can delineate between hypomorphic and full loss‐of‐function effects for a given protein, which could be useful when interpreting and prioritizing variants or patient genotypes.

Some heterogeneity in predictor performance is not surprising. Previous efforts to benchmark their accuracy in reproducing realistic ΔΔG values have revealed highly varied performance, owing to the different methodological approaches and biases. A likely reason for the success of FoldX and Rosetta are their empirical scoring functions, containing energetic and statistical terms parametrized based on experimental data. It is unlikely that the source of high FoldX and Rosetta performance is overtraining on test data, as we are benchmarking a different kind of performance altogether. Instead, the high agreement is likely due to an underlying association of loss‐of‐function mechanisms with the protein and phenotype in question. RaSP (Blaabjerg et al., 2023), a deep learning‐based method that simplifies structural protein representations, benefits indirectly through being parametrized against Rosetta predictions, while leveraging considerably improved computational speed. More novel approaches combining machine learning with sequence‐ or structure‐based features, such as mCSM, DDGun3D, PoPMuSiC and INPS3D, do not seem to overall be as effective at ranking functional impacts. However, these state‐of‐the‐art methods have been shown to be accurate at predicting actual ΔΔG values, and improve upon other methodologies in terms of prediction symmetry, and are less sensitive to resolution and substitution type biases (Caldararu et al., 2021; Montanucci, Savojardo, et al., 2019; Pucci et al., 2018; Sanavia et al., 2020; Usmanova et al., 2018).

Interestingly, we observed the more unconventional methods, such as ENCoM and DynaMut2, benefit the most from using absolute ΔΔG values, but also, despite having adjusted for effect directions of predictors and DMS sets to match, often demonstrate moderate inverse correlations, likely due to experimental noise. It is also important to point that DDGun3D and INPS3D, the hybrid sequence and structure‐based approaches, effectively include sequence‐derived features representing evolutionary conservation in their predictions. Conservation is known to be predictive of damaging mutations, regardless of molecular mechanism, which would suggest that these methods could be capable of predicting mutations at conserved positions to be more (de)stabilizing than they actually are, resulting in a stronger correlation with the functional scores from DMS assays. Given that non‐loss‐of‐function disease variants tend to be structurally milder, we would expect them to be poorly predicted by conventional stability predictors, since their damaging effects are unlikely to be due to destabilization (Gerasimavicius et al., 2022). DDGun3D and INPS3D appear to show relatively better correlations, compared with other methods, on essential, highly conserved genes, such as HSP82 and PAB1, or some genes with mixed disease mechanism like TARDBP and SRC (Abe et al., 2019; Kabashi et al., 2010; Turro et al., 2016). However, sequence features do not appear to consistently favor the prediction of non‐LOF disease gene variants, like CALM1, and overall, these hybrid predictors demonstrate relatively poor performance in the correlation ranking, and especially on the abundance‐based DMS datasets.

Interestingly, we also saw a marked performance increase when combining FoldX and Rosetta predictions into an ensemble scoring approach we dubbed “Foldetta,” which is simply the mean prediction value of the two tools for a given variant. Despite observing that FoldX and Rosetta can give very different evaluations of a variants' effect, going as far as producing stability values of opposing signs for the same mutation, the ensemble score correlates better with DMS data, matching the performance of VEPs and even substantially outperforming them when only ranking methods on abundance‐based datasets. However, modern multi‐feature methods like DDGun3D, INPS3D and DynaMut2, which themselves combine the methodologies or even direct predictions from other tools (original version of DynaMut) do not seem to demonstrate the same capabilities. This demonstrates that the noise in current computational stability predictors could be alleviated by combining multiple well‐performing tools, perhaps with a specific emphasis on methodologies based on empirical potentials that approximate physics terms, as both FoldX and Rosetta fall into this category. Overall, the performance of Foldetta suggests stability predictors have applicability in disease variant identification, and further ensembling approaches combining currently available predictors may lead to yet even better results, especially if ensembles are trained and the individual tools are weighted.

In this study we also demonstrated the utility of using complex structures of biological units, as monomer structures may not be sufficient for assessing the full functional impact of a variant, for instance due to molecular mechanisms involving intermolecular interactions, as shown previously (Gerasimavicius et al., 2022). Both FoldX and Rosetta, the best ranked methods, and ENCoM, the poorest, were able to take into account structures containing more than one protein chain, and in the case of FoldX and Rosetta also other biomolecules. All methods saw increased correlations with DMS values for some datasets which are based on phenotypes that involved assessing binding, aggregation, or for genes with known involvement in functional interactions. The correlation between FoldX and the transcription factor GAL4 saw considerable benefit, because FoldX is able to evaluate the stability perturbations involving DNA. Of course, such an approach depends on having available structures, and being able to assess the relevance of a given assembly for assessing a particular phenotype. While DeepMind have now made monomeric AlphaFold2 model structures of the whole proteome available to all, the Protein Data Bank still remains the main source of functionally relevant protein complexes.

Our work hints at the pervasiveness of destabilizing loss‐of‐function mechanisms throughout the functional variant landscape, and the importance of choosing the most informative phenotypes for DMS experiments. Both stability predictors and VEPs we tested performed the best on experiments interrogating protein abundance and stability, such as various assays involving fluorescent reporters or VAMP‐seq, and some antibiotic survival experiments. Importantly, however, not all loss‐of‐function missense mutations will be due to protein destabilization: for example, they may impact functionally important sites or perturb protein allostery (Clarke et al., 2016). Moreover, gain‐of‐function and dominant‐negative mechanisms are also common pathogenic mechanisms (Backwell & Marsh, 2022). In these cases, both stability predictors and abundance‐based DMS experiments will be less likely to accurately reflect the biologically important impacts of variants.

In the case of proteins with multiple functions or disease mechanisms, multiple different assays would be required to gleam the full scope of a variant. This could be alleviated by using competitive growth assays, which should capture the broadest selection of functional effects from variants; although, the delineation of what molecular mechanism might be responsible for the increase or reduction in fitness is lost. Other general issues with DMS data are that it can be noisy, restricted to a small set of experimental conditions, and also removed from the original cellular context (Chiasson et al., 2019). Due to these limitations, as well as the enormous resource cost of most current DMS methodologies, they are unlikely to replace computational prediction tools as the main avenue to fully understanding functional effects of missense mutations in the near future. However, an exciting new methodology, dubbed cDNA display proteolysis, was recently shown to be capable of assessing functional variant effects on protein thermodynamic stability at tremendous scale and speed (Tsuboyama et al., 2022). While limited to a stability phenotype, such a DMS approach also presents a valuable opportunity to gleam insight into the mechanisms of LOF disease, further test the accuracy of current computational tools on a large independent dataset and use it for training and developing better methodologies.

## 4 METHODS

### 4.1 Structural DMS variant dataset collection and mapping

Starting from the set of DMS datasets we compiled as part of our recent VEP benchmarking studies (Livesey & Marsh, 2020; Livesey & Marsh, 2022b), we also added additional datasets gathered from experimental research publications and MaveDB (Esposito et al., 2019). All datasets are listed in Table S1, along with publication references or MaveDB accession codes.

Protein Data Bank structures were selected through a procedure published previously (Gerasimavicius et al., 2022), choosing the first biological assembly for each structure as representative of the biologically relevant quaternary structure. The mutation mapping pipeline has been previously described (Gerasimavicius et al., 2022). Protein chains with more than 90% sequence identity to a human protein over a region of at least 50 amino acid residues were considered. Mutations were only mapped to nonhuman structures in cases where the residue and its adjacent neighbors were the same as the human wild‐type sequence. Including nonhuman structures with this approach allowed us to substantially increase the size of our dataset. Structures with best resolution followed by largest biological assembly were prioritized for mapping in the case of multiple available structures for a residue. For PDB files containing multiple occupancies of a single residue, only the first occurring entry was selected and residues missing from PDB structures were not considered. For NMR ensembles the first frame was chosen.

Variants that could not be mapped to PDB structures were evaluated on AlphaFold2 models (Jumper et al., 2021). AlphaFold models were accessed and downloaded on July 27, 2021 from https://alphafold.ebi.ac.uk. The DMS variants were mapped to AlphaFold models based on UniProt sequence positions.

### 4.2 Structure‐based variant stability predictors and variant effect predictors

FoldX 5.0 was run using default parameters as previously described (Delgado et al., 2019; Gerasimavicius et al., 2022). Both protein subunit and complex structures were used where available to also take into account intermolecular interactions. The structures were passed through the “RepairPDB” function prior to ΔΔG calculations. DDGun (Montanucci et al., 2022) source code was downloaded from https://github.com/biofold/ddgun and run using the 3D protocol. ENCoM (Frappier et al., 2015) was run both on monomeric and complex PDB protein structures throughout two separate instances, source code was downloaded from https://github.com/NRGlab/ENCoM. The Cartesian ΔΔG application from Rosetta suite (Linux build 2021.16.61629) was run based on the protocol laid out in Park et al. (2016), while using the Ref2015 scoring function. The structures were relaxed according to the protocol for both monomeric and complex PDB structures. Results from three prediction iterations were averaged and ΔΔG values were derived by taking the difference between the wild‐type and mutant values for each individual run. For complex evaluation, PDB structures 4JZW and 4JZZ were modified to introduce cysteines instead of some nonstandard residues for Rosetta to recognize the disulfide bonds. RaSP (Blaabjerg et al., 2023) was run on a modified Google Colab notebook, based on a mix of code from GitHub commits 26b0b1a and 518624e. INPS3D (Savojardo et al., 2016), mCSM (Pires et al., 2014), DynaMut2 (Rodrigues et al., 2021), and PoPMuSiC (Dehouck et al., 2011) webservers were queried in Python 3.8.8 using RoboBrowser (https://github.com/jmcarp/robobrowser) or Selenium (https://github.com/baijum/selenium-python) packages. Some predictions for certain datasets or variants could not be completed due to software or webserver errors.

The Foldetta stability score was derived as a mean of FoldX and scaled Rosetta predictions for each variant. As Rosetta predictions are initially produced in REUs (Rosetta Energy Units), they need to be divided by a scaling factor of 2.94 (previously established in Park et al. (2016) to bring them onto the kcal/mol scale as other predictors, like FoldX. In the case of absolute stability values, the mode of the FoldX and Rosetta predictions was taken before combining them into Foldetta.

For DMS datasets present in our recent benchmarking studies, we used the previously calculated VEP values (Livesey & Marsh, 2020; Livesey & Marsh, 2022b). For newly included DMS datasets, VEP values were obtained using the same pipeline. Where available, VEP predictions were obtained using the dbNSFP database version 4.0 (Liu et al., 2020). Further predictor scores were obtained from predictor web‐interfaces like SNAP2 (Hecht et al., 2015), or run locally for EVcouplings (Hopf et al., 2019) SIFT(Ng & Henikoff, 2003), and DeepSequence (Riesselman et al., 2018).

To compare the agreement between GAL4 DMS scores and FoldX stability predictions for variants, both score sets were rank normalized to the range of 0–1. As they showed better correlation, absolute stability values were chosen for the comparison, and any variants that were indicated to increase the functional outcome of the DMS assay were removed to focus on the loss‐of‐function aspect of the predictions. Normalized stability values were subtracted from the normalized DMS scores, where a positive delta for a variant indicates FoldX is underpredicting its functional effect, in relation to DMS data, while a negative delta means FoldX overestimates the functional impact of a variant. A value close to zero indicates agreement between FoldX and the DMS assay on the relative severity of a variant. To visualize prediction accuracy on a structure, median delta values were derived from all variants with a DMS score at a given position.

### 4.3 Statistical analyses

Full‐pairwise Spearman's correlations for complete observations were calculated using the R “psych” (Revelle, 2022) and “corrplot” (Wei et al., 2021) packages. The directions of the DMS scores and stability predictor values were adjusted to match a single “direction.”

For the predictor correlation ranking against DMS functional values, a scoring scheme involving pairwise comparisons of each predictor against every other predictor for every DMS dataset was derived. For a given DMS dataset and two predictors, only the common subset of variants, for which both prediction and assay values were available, was used to calculate Spearman's rho values. The correlation values were rounded to two digits after the decimal point and were compared only if neither predictor was completely missing data for a given DMS dataset. After a successful comparison, the predictor with a larger correlation value was rewarded a point, or each predictor was granted half a point in the case of a draw after rounding. In the end, each predictor's score was divided by the total number of successful comparisons it was involved in to normalize the scores. This normalization also gives a chance for predictors with a few missing DMS datasets not to fall behind in the scoring scheme due to undersampling issues. Error bars were derived using the “binom.confint” function from the “binom” package (Dorai‐Raj, 2014).

## CONFLICT OF INTEREST STATEMENT

The authors declare no conflicts of interest.

## Supporting information


**Data S1:** Supporting information

## Data Availability

All the DMS dataset variants, structure identifiers used in the dataset, and stability predictor values are available at https://www.doi.org/10.17605/OSF.IO/ZTV8A.
